# Fibroblast Activation Protein (FAP)-Mediated Cleavage of Type III Collagen Reveals Serum Biomarker Potential in Non-Small Cell Lung Cancer and Spondyloarthritis

**DOI:** 10.3390/biomedicines12030545

**Published:** 2024-02-29

**Authors:** Rasmus S. Pedersen, Jeppe Thorlacius-Ussing, Maria G. Raimondo, Lasse L. Langholm, Georg Schett, Andreas Ramming, Morten Karsdal, Nicholas Willumsen

**Affiliations:** 1 Nordic Bioscience A/S, 2730 Herlev, Denmarklla@nordicbio.com (L.L.L.); mk@nordicbio.com (M.K.); nwi@nordicbio.com (N.W.); 2 Department of Biomedical Sciences, University of Copenhagen, 2200 Copenhagen, Denmark; 3 Deutsches Zentrum Immuntherapie, University Hospital Erlangen, Friedrich-Alexander University Erlangen-Nürnberg, 91054 Erlangen, Germany; mariagabriella.raimondo@uk-erlangen.de (M.G.R.); georg.schett@uk-erlangen.de (G.S.); andreas.ramming@uk-erlangen.de (A.R.); 4 Department of Internal Medicine 3-Rheumatology and Immunology, University Hospital Erlangen, Friedrich-Alexander University Erlangen-Nürnberg, 91054 Erlangen, Germany

**Keywords:** fibroblast activation protein (FAP), extracellular matrix remodeling, serum biomarker, non-small cell lung cancer (NSCLC), spondyloarthritis

## Abstract

Fibroblast activation protein (FAP) is a known promoter of tumor development and is associated with poor clinical outcome for various cancer types. Being specifically expressed in pathological conditions including multiple types of fibrosis and cancers, FAP is an optimal target for diagnostics and treatment. Treatment strategies utilizing the unique proteolytic activity of FAP are emerging, thus emphasizing the importance of biomarkers to directly assess FAP activity. FAP is a type II transmembrane serine protease that has been shown to cleave collagens and other ECM components. In this study, we developed an ELISA assay (C3F) targeting a circulating type III collagen fragment derived from FAP cleavage to reflect FAP activity. We demonstrated that C3F was specific to the neoepitope of the cleavage site and that the fragment was generated through FAP cleavage of type III collagen. We measured C3F in serum from a cohort of patients with non-small cell lung cancer (NSCLC) (*n* = 109) matched to healthy subjects (*n* = 42) and a cohort of patients with spondyloarthritis (SpA) (*n* = 17) matched to healthy subjects (*n* = 19). We found that C3F was significantly elevated in patients with NSCLC and in patients with SpA compared to healthy controls (*p* < 0.0001 and *p* = 0.0015, respectively). These findings suggest that C3F is a promising non-invasive biomarker reflecting FAP activity, which may aid in understanding tumor heterogeneity and potentially FAP-targeted therapies.

## 1. Introduction

Fibroblast activation protein-α (FAP) is a type II transmembrane serine protease primarily expressed during pathologies such as cancer, fibrosis, and arthritis [1,2,3,4]. While cancer-associated fibroblasts (CAFs) are often associated with high levels of FAP in cancer, multiple other cell types have been shown to have high FAP expression during various pathological conditions, including synovial fibroblasts, myo-fibroblasts, and chondrocytes [4,5,6,7].

FAP may act on extracellular matrix remodeling and the regulation of intracellular signaling, angiogenesis, the epithelial-to-mesenchymal transition, and immunosuppression, and therefore is associated with clinical outcome. FAP has been shown to promote tumor progression and to be a predictor of poor overall survival for multiple cancer types, including colon cancer, prostate cancer, pancreatic ductal adenocarcinoma, ovarian cancer, and NSCLC [4,8,9,10,11,12,13].

The specificity of FAP to pathological conditions makes it an optimal target for diagnostics and the treatment of diseases such as cancer, and its potential is being investigated through multiple approaches [14,15,16,17,18,19,20,21,22,23]. Recent studies are utilizing this concept by using radiolabeled FAP inhibitors (FAPIs) as tracers for PET imaging and have shown the potential for the detection of both primary tumors and metastasis that were undetectable with ^18^F-FDG tracers for multiple types of cancers [14,15,16]. Besides the diagnostic outcome, the specificity and sensitivity of the FAPI tracer could potentially contribute to the better outlining of target areas for radiotherapy [16,17]. Other studies have investigated the potential of using FAP as a target for cancer treatments with different strategies including cancer vaccines, FAP-specific CAR-T cell treatment, and bispecific antibody-guided immunotherapy [18,19,20,23].

FAP is part of the prolyl peptidase family and has dipeptidyl peptidase activity similar to dipeptidyl peptidase-4 (DPP4) [2]. However, in addition to dipeptidyl peptidase activity, FAP also has a unique endopeptidase activity allowing FAP to cleave with a preference for proline as P1 and glycine as P2 without being limited to the N-terminus of the peptide or protein [5,24]. Some studies have shown the potential of utilizing the combination of the disease-specific expression of FAP and the unique endopeptidase activity for drug-delivery systems by having an inactive pro-drug that, with a FAP-specific cleavage site that leads to the release of an active chemotoxin when cleaved, thereby activating the drug only in the diseased areas as these are the ones that will have FAP activity [21,22].

To catalyze successful drug development of drugs targeting or utilizing FAP activity and to ensure the optimal treatment and patient care in the clinic for patients receiving these treatments, biomarkers for the regular quantification of FAP activity instead of protein expression or amount are needed for optimal patient stratification and treatment effect monitoring. A recent and promising tool is the use of probes or FAPIs, of which there are various types, but they are either based on either FAP-specific binding or FAP-specific cleavage, resulting in trapping or releasing a detectable signal (e.g., fluorophore or radiotracer). While these approaches show great potential in locating the presence and activity of FAP for potential diagnostics and drug testing, they are expensive procedures [25,26,27].

FAPs are involved in the remodeling of the ECM regarding both structure and composition [28,29]. FAPs have been shown to cleave multiple ECM components including type I, type III, and type V collagen. However, as FAP cannot cleave triple helical collagens, pre-digesting, e.g., with matrix metalloproteinases (MMPs), or denaturation by heating is needed to allow FAP cleavage [5,30].

Type III collagen is primarily derived from activated fibroblasts and increased formation has been shown for CAFs [31,32]. The increased expression, maturation, and remodeling of type III collagen have been associated with cancer and multiple fibrotic disorders [31,33,34,35]. Multiple MMPs have been shown to cleave native type III collagen including MMP-1, MMP-8, MMP-9, MMP-12, and MMP-13 [33,36,37,38]. Zhang et al. have investigated FAP cleavage in the ECM using degradomics based on terminal amine isotopic labelling of substrates (TAILS) in mice and identified novel cleavage sites that could potentially be used to reflect FAP activity [24].

In this study, we developed and validated an ELISA targeting an FAP-generated cleavage fragment of type III collagen as a novel biomarker to non-invasively assess FAP activity. We hypothesized that this fragment would be detectable in human serum with elevated values for pathological conditions with association to FAP expression. We proceeded to measure this biomarker in a cohort of patients with non-small cell lung cancer (NSCLC), and spondyloarthritis (SpA) demonstrated biological relevance.

## 2. Materials and Methods

### 2.1. Target Identification

Zhang et al. identified various potential cleavage sites for multiple substrates of FAP in the ECM in mice [24]. From these sites, we chose the human equivalent to ^1069^AGPSGAPGPA of mice type III collagen alfa 1 chain as the antibody target: ^1070^AGPAGAPGPA (C3F). We used BLAST on the amino acid sequence to identify potential cross-reaction to other human proteins based on homology using NPS@ (Network Protein Sequence Analysis) with UniportKB/Swiss-prot database. From these three sequences, the following were deemed as relevant potential off-targets for the antibody: AGPAGAAGQP (from alpha-2 chain of collagen type I), PGPAGAPGPA (from alpha-2 chain of collagen type V), and AGPQGAPGPA (from alpha-1 chain of collagen type II).

### 2.2. Antibody Development and Specificity

For production of antibodies towards the target sequence ^1070^AGPAGAPGPA^1079^, the immunogenic peptide was produced by binding keyhole limpet hemocyanin (KLH) carrier protein to the 10aa peptide, with glycine and cysteine residues incorporated at the C-terminal to ensure proper binding (AGPAGAPGPA-GGC), through covalent cross-linking using sulfosuccinimidyl 4-(N-maleimidomethyl) cyclohexane-1-carboxylate, SMCC (Thermo Scientific, Waltham, MA, USA, cat. #22322). We immunized six-week-old Balb/C mice subcutaneously with 200 µL of an emulsified antigen, consisting of 100 µg of the immunogenic peptide mixed with Sigma Adjuvant System (Sigma-Aldrich, St. Louis, MO, USA, cat. #S6322) and Specol (Invitrogen, Waltham, MA, USA, cat. #7925000). The immunization was repeated at 2-week intervals until stable levels of serum titer was achieved. The mouse with the highest titer was then rested for four weeks before receiving intravenous injection of a boost of 100 µg of the immunogenic peptide in 100 µL of 0.9% NaCl solution. Spleen cells were fused with SP2/0 myeloma cells as described by Gefter et al. [39] to create hybridoma cells. These hybridoma cells were then cultured in 96-well microtiter plates, using standard limited dilution to secure monoclonal growth. Monoclonal antibodies were purified from the supernatant using protein-G-columns (GE Healthcare Life Sciences, Little Chalfont, UK, cat. #17-0404-01) in accordance with manufacturer’s instructions.

Antibody specificity towards the targeted neoepitope amino acid sequence (selection peptide (AGPAGAPGPA)) was demonstrated by using the competitive ELISA with biotinylated selection peptide (AGPAGAPGPA-K-Biotin) as the coater and a two-fold dilution series of 10- and 30-amino-acid selection peptides (AGPAGAPGPA and AGPAGAPGPAGSRGAPGPQGPRGDKGETGE, respectively), elongated peptide (PAGPAGAPGPA), truncated peptide (_GPAGAPGPA), and three deselection peptides from the potential off-targets previously described: (AGPAGAAGQP, PGPAGAPGPA, and AGPQGAPGPA). All used peptides were purchased from Genscript (Piscataway, NJ, USA).

In addition, we incubated recombinant type III collagen (Sigma-Aldrich, St. Louis, MO, USA, cat. #CC054) with MMP9 (Bio-Techne, Minneapolis, MN, USA, cat. #911-MP) or MMP13 (bio-techne cat. #511-MM) for 24 h and stopped the reaction with EDTA. The type III collagen incubated with MMP13 was then incubated with or without FAP (Bio-Techne, Minneapolis, MN, USA, cat. #3715-SE) for another 24 h followed by measurement of C3F to demonstrate that FAP-mediated cleavage of type III collagen could be measured in the assay.

### 2.3. Assay Development and Evaluation

The technical performance of the nordicC3F assay was evaluated through the following validation steps: determination of quantification range, inter- and intra-assay variation, dilutional linearity, spiking accuracy, analyte stability, interference tolerance of hemoglobin, lipids, and biotin.

The lower limit of quantification (LLOQ) was assessed based on five independent runs each with six replicates of four different serum samples with C3F biomarker levels around the expected lower limit of measurement and CV% values around 20%. A power regression model was made from the C3F biomarker levels and CV% of these samples, and LLOQ was defined as the C3F biomarker levels resulting in a CV% of 20% based on this model. The upper limit of quantification (ULOQ) and the inter- and intra-assay variations were estimated from 10 independent runs of the assay with the 10 samples in duplicates. The samples consisted of five human serum samples and four human serum samples spiked with a 30-amino-acid selection peptide and one human urine sample. ULOQ was defined as the value of the highest point on the standard curve with CV% and RE% below 20%. The intra-assay variation was defined as the average CV% between sample double-determinations for each run, while the inter-assay variation was defined as the average CV% of the 10 samples between runs.

To test dilution recovery, four human serum samples were serially diluted 2-fold and the RE% relative to previous dilution steps was calculated. A minimal required dilution (MRD) was defined based on the least diluted dilution step from which further dilutions had an RE% between 80 and 120%.

Spike-in accuracy was investigated both by spiking three serum samples with high C3F values into three serum samples with low C3F values followed by calculation of the RE% relative to the expected concentration.

Three human serum samples were thawed and frozen zero, one, two, three, four, or five times before being measured in the C3F assay. RE% was calculated relative to the samples that were not thawed and frozen prior to measurement.

Hemoglobin, biotin, and lipids are known to be interfering substances; therefore, it was investigated whether these can influence the assay by spiking human serum samples with these substances in both low and high concentrations (hemoglobin: low = 2.5 ng/mL, high = 5 ng/mL; biotin: low = 5 ng/mL, high = 100 ng/mL; lipid: low = 1.5 ng/mL, high = 5 ng/mL). The interferences were calculated as percentage recovery of spiked samples with non-spiked samples as reference.

### 2.4. C3F Assay Protocol

The C3F assay underwent extensive optimization regarding assay buffer, incubation time, temperature, antibody/coater peptide ratio, and conjugation of horseradish peroxidase (HRP) to the antibody. This optimization led to the final protocol where 96-well streptavidin-coated plates were coated with 100 µL of 3.5 ng/mL biotinylated selection peptide (AGPAGAPGPA-K-biotin) dissolved in assay buffer (25 mM phosphate-buffered saline, 1% bovine serum albumin (*w*/*v*), 0.1% Tween-20 (*w*/*v*), 2 g NaCl/L, pH 8.0) followed by incubation in darkness at 20 °C for 30 min while shaking with 300 revolutions per minute (rpm). The plates were then washed 5 times with washing buffer (20 mM TRIS, 50 mM NaCl, pH 7.2). After washing, 20 µL of a 2-fold serial dilution of selection peptide (AGPAGAPGPAGSRGAPGPQGPRGDKGETGE) starting at 500 nM as well as 20 µL of 1:2-diluted serum samples were added to the plate followed by the addition of 100 µL of 12.5 µL C3F-targeting antibody dissolved in assay buffer, followed by incubation at 4 °C in darkness while shaking (300 rpm). After 20 h (±1 h), the plates were washed 5 times in washing buffer followed by the addition of 100 µL of secondary HRP-antibody (Rabbit anti-mouse IgG, Jackson, Ely, Cambridgeshire, UK) (Cat. #315-035-045) diluted in assay buffer to a final dilution of 1:6000 and incubated at 20 °C for 1 h while shaking (300 rpm). The plates were washed 5 times in washing buffer again and 100 µL TMB (Kem-En-Tec Diagnostics (Cat. #4380)) was added to the plates followed by 15 min of incubation in darkness at 20 °C while shaking (300 rpm). After the incubation, the reaction was stopped by adding 100 µL of 1% sulfuric acid. The plates were analyzed using VersaMax ELISA microplate reader (Molecular Devices, San Jose, CA, USA) at 450 nm, with 650 nm as reference. A standard curve was generated using SoftMax Pro (Version 7.1) and the data were analyzed using GraphPad Prism (version 9).

### 2.5. Assessment of MMP Degraded Type III Collagen

The biomarker nordicC3M (cat. No 1200-03) was measured to reflect MMP-degraded type III collagen in human serum samples via ELISA. C3M was measured according to the manufacturer’s instructions (Nordic Bioscience A/S, Herlev, Denmark).

### 2.6. Subjects

The biomarker C3F was measured in serum samples from patients with NSCLC (*n* = 109) and sex- and age-matched healthy individuals (*n* = 42) (cohort 1). Patient demographics are outlined in Table 1 and include age, sex, body mass index (BMI), NSCLC subtype, and stage (American Joint Commission on cancer, 8th edition). All serum samples from patients with NSCLC cancer were obtained from Proteogenex (Los Angeles, CA, USA). The sample collection was obtained after patients gave their informed consent and with the approval by the Russian Oncological Research Centre n.a. Blokhin RAMS (PG-ONC 2003/1) (Moscow, Russia) and the Western Institutional Review Board, Inc. (Puyallup, WA, USA) (WIRB^®^Protocol #20161665). Control serum samples from healthy individuals were obtained from the commercial vendor BioIVT (Westbury, NY, USA). BioIVT (Westbury, NY, USA) has an appropriate institutional review board/independent ethical committee-approved sample collection. All investigations were carried out in accordance with the Helsinki Declaration.

Furthermore, C3F was measured in serum samples from a small cohort (cohort 2) of patients with SpA (*n* = 17) and compared with a reduced version of the healthy individuals from cohort 1 (*n* = 19) to make it sex- and age-matched. The selection of these healthy individuals was based on sex and age alone, blinded for measured C3F levels.

Patient demographics are outlined in Table 2 and include age, sex, disease subtype, and whether the patients had received treatment or not.

Written informed consent was obtained from all patients for the evaluation and publication of their anonymized data. Subjects with axial spondyloarthritis (*n* = 7) fulfilled the ASAS criteria [40].

### 2.7. Statistics

Normal distribution of C3F biomarker levels within the groups was evaluated based on QQ-plot and D’Agostino’s K^2^ test. Biomarker levels in serum from patients with NSCLC or SpA and healthy individuals were compared using the Mann–Whitney test. The diagnostic potential of C3F was further investigated using Wilson/Brown Receiver operating characteristic (ROC) curve analysis. *p*-values below 0.05 were considered statistically significant.

GraphPad Prism software (version 9) was used for statistical analyses and graphic designs.

## 3. Results

### 3.1. Technical Evaluation of the C3F ELISA Assay

The ELISA for the quantification of C3F underwent a technical evaluation to demonstrate its quality. The lower limit of detection was determined to be 0.96 nM and the quantification range (LLOQ-ULOQ) of the C3F assay was determined to range from 1.27 to 250 nM (2.54 to 500 nM corrected for 1:2 dilution). The mean dilution recovery in serum from undiluted to 1:2 dilution and from 1:2 dilution to 1:4 dilution was 133% and 93%, respectively. Based on the dilution recovery, the minimum required dilution was determined to be 1:2. The mean spiking recovery from spiking serum in serum was 90%. The average analyte recovery for interference of low and high concentrations was 102% and 106% for hemoglobin, 107% and 109% for biotin, and 103% and 108% for lipids, respectively. The inter- and intra-assay variations were 13% and 7%, respectively. Analyte recovery in human serum was 84% after four freeze/thaw cycles. Collectively, this shows that the ELISA assay is robust and unaffected by common serological interferents. The results from the technical evaluation are summarized in Table 3.

### 3.2. C3F Antibody Binding Is Specific to the FAP-Cleaved Type III Collagen Fragment

**Figure 1:**
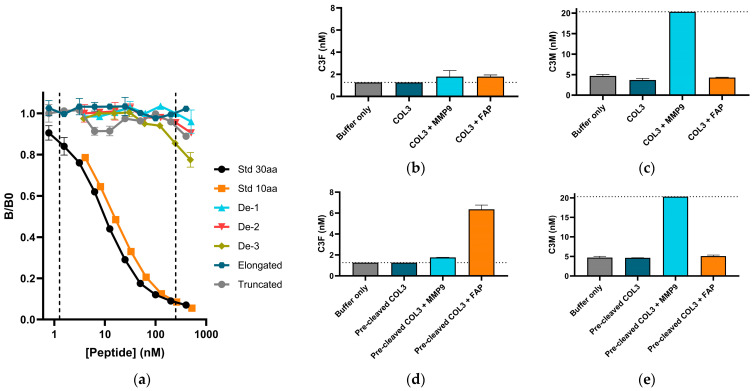
Assay specificity: (**a**) Standard curves of signal inhibition relative to background (B/B0) with increasing peptide concentrations for 30aa selection peptide (std) (AGPAGAPGPAGSRGAPGPQGPRGDKGETGE), 10aa selection peptide (AGPAGAPGPA), deselection peptide (De) 1, 2, and 3 (AGPAGAAGQP, PGPAGAPGPA, and AGPQGAPGPA, respectively), elongated peptide (PAGPAGAPGPA), and truncated peptide (_GPAGAPGPA). Dotted lines indicate measurement range. (**b**,**c**) Biomarker measurements of C3F (**b**) and C3M (**c**) in solution of recombinant type III collagen (COL3) after incubation with/without matrix metalloproteinases 9 (MMP9) or fibroblast activation protein (FAP), with buffer as control. (**d**,**e**) Biomarker measurements of C3F (**d**) and C3M (**e**) in solution of recombinant type III collagen pre-cleaved with MMP13 (pre-cleaved COL3) followed by incubation with/without MMP9 or FAP, with buffer as control. Dotted lines indicate lower limit measurement range (LLMR) for C3F (**b**,**d**) and upper limit measurement range (ULMR) for C3M (**c**,**e**).

To demonstrate the specificity of the C3F assay towards the targeted amino acid sequence corresponding to the N-terminus of the C-terminal type III collagen fragment generated from FAP-mediated cleavage between amino acid residues P^1069^ and A^1070^, the inhibition properties of one amino acid truncated and elongated versions of the selection peptide were investigated. No inhibition of the signal was seen within the measurement range from any of the deselection peptides, or the elongated or truncated peptide, demonstrating that the assay only detected the target sequence when A^1070^ was presented as the terminal (neo-epitope specificity), thus indicating that the assay was specific towards the cleavage fragment (Figure 1a).

To show that the C3F fragment is generated through FAP cleavage of type III collagen, we performed in vitro cleavage with FAP and MMP9 as a control. While we confirmed that MMP9 cleaved native type III collagen by quantification of C3M, C3F could not be generated from native type III collagen (Figure 1b,c). To enable FAP cleavage, type III collagen was denatured from incubation with MMP13, an MMP known to have collagenase activity, prior to FAP or MMP9 cleavage. The C3F fragment was only generated from FAP cleavage of the pre-cleaved type III collagen and not from MMP9 cleavage or the MMP13 pre-cleavage (Figure 1d,e). To our knowledge, this is the first confirmation of the presence of this FAP cleavage site in humans.

Together, this demonstrates that the ELISA assay can reflect FAP activity by specifically quantifying the C3F fragment.

### 3.3. C3F Is Elevated in Serum from Patients with NSCLC

**Figure 2:**
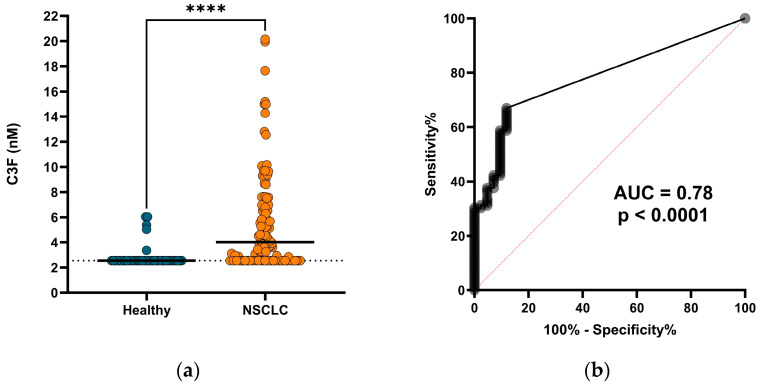
Diagnostic potential of C3F for patients with NSCLC: (**a**) C3F biomarker levels in serum from healthy subjects (*n* = 42) and patients with NSCLC (*n* = 109). Black lines indicate median values, dotted lines indicate LLMR (adjusted for dilution). **** indicates *p* < 0.0001. (**b**) Receiver operating characteristic (ROC) curve for patients with NSCLC and healthy subjects described in (**a**). Area under the curve (AUC) and *p*-value are shown.

Having shown that C3F could be generated in vitro, we moved on to investigate the biological relevance by measuring the biomarker in serum from a cohort of 109 NSCLC patients and 42 age- and sex-matched healthy individuals. A total of 37 (88%) of the healthy subjects and 36 (33%) of the patients with NSCLC had C3F levels below the measurement range. Yet, we saw that C3F was significantly elevated in the serum from patients with NSCLC (median = 4.02 nM, range: 2.54–20.2 nM) compared to serum from the healthy individuals (median = 2.54 nM, range: 2.54–6.04 nM) (*p* < 0.0001) (Figure 2a). In this study, the C3F biomarker could significantly discriminate between patients with NSCLC and healthy individuals with an area under the receiver operating characteristic curve (AUROC) of 0.78 (*p* < 0.0001) (Figure 2b), indicating a diagnostic potential for C3F as a biomarker.

### 3.4. C3F Levels Are Elevated in Patients with SpA

**Figure 3:**
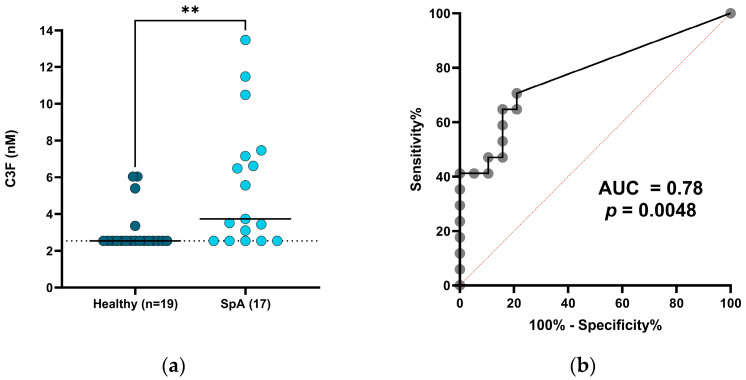
Diagnostic potential of C3F for patients with SpA: (**a**) C3F biomarker levels in serum from healthy subjects (*n* = 19) and patients with SpA (*n* = 17). Black lines indicate median values, dotted lines indicate LLMR (adjusted for dilution). ** indicates *p* < 0.01. (**b**) ROC curve for patients with SpA and healthy subjects described in (**a**). AUC and *p*-value are shown.

To further evaluate the clinical relevance of C3F in non-cancerous diseases, we measured the biomarker in a small cohort of serum samples from patients with SpA (*n* = 17) and age- and sex-matched healthy controls (*n* = 19) (cohort 2). Similar to patients with NSCLC, we found that C3F was significantly elevated in serum from patients with SpA (median = 3.73 nM, range = 2.54–13.47 nM) compared to serum from healthy controls (median = 2.54 nM, range = 2.54–6.04 nM) (*p* = 0.0015) (Figure 3a). For this cohort, C3F could significantly discriminate between healthy controls and patients with SpA with an AUC of 0.78 (*p* = 0.0048) (Figure 3b).

## 4. Discussion

In this study, we utilized this unique endopeptidase activity of FAP to develop an ELISA targeting a specific FAP-cleaved type III collagen fragment (C3F) to serologically reflect FAP activity. To our knowledge, this is the first FAP-activity-assessing assay utilizing a naturally occurring peptide fragment. We demonstrated that the assay was neo-epitope-specific and dependent on the FAP cleavage of pre-cleaved type III collagen. The dependency of pre-cleaved type III collagen suggests that C3F is preferably generated in tissue with high turnover and disease activity, potentially furthering the disease specificity of the biomarker. We showed that C3F could be detected in serum and saw elevated levels of C3F for both patients with NSCLC and SpA compared to healthy subjects, thus demonstrating a novel association between serum levels of C3F and the two diseases, indicating a diagnostic biomarker potential of C3F for FAP-related diseases.

Despite multiple studies showing FAP to be involved in tumor progression, the exact role of FAP in the TME is not well understood. With most studies addressing FAP by mRNA or protein expression, the role of FAP protease activity remains mostly unexplored [29,41]. However, the protease activity of FAP has been shown to be directly associated with tumor growth [42]. With ECM remodeling already being known as an important process in tumor progression, more knowledge of the precise role of the protease activity of FAP in the TME could be key to better understanding the disease and treatment strategies [43]. By quantifying FAP-cleaved type III collagen, we believe that the C3F biomarker assay could be a tool for investigation of the role of FAP in ECM remodeling.

The C3F ELISA presented in this study has the advantages of targeting a fragment that is dependent on FAP cleavage, meaning that everything that is measured is FAP activity in contrast to existing ELISAs that measure the full FAP protein level [44]. However, as the targeted fragment originates from type III collagen, the amount and availability of type III collagen in proximity to FAP also influence the quantity of the fragment and therefore the C3F measurements. As both FAP and type III collagen are known to be associated with myo-fibroblast or CAF activity, the C3F biomarker may also be a proxy for specific fibroblast/CAF activity given that CAFs are highly heterogeneous in both function and expression profile and are often divided into multiple subtypes based on their expression of alpha smooth muscle actin (αSMA) and fibroblast activation protein (FAP) [45,46,47,48,49].

While we could significantly distinguish patients with NSCLC and SpA from healthy controls in cohort 1 and 2, respectively, most of the healthy controls showed C3F levels below the measurement range. This could either be due to the technical properties of the assay or reflect the fact that this fragment is not actively generated in healthy individuals and is only generated in a fraction of patients with disease. In support of the latter, FAP cleavage of type III collagen, and therefore the generation of C3F, requires that the collagen is denatured or pre-cleaved [5,30], and this phenomenon is often associated with diseases such as cancer and arthritis and not seen in healthy homeostatic tissues [50,51,52,53].

In contrast to PET/CT scans with FAPI-tracers, the C3F ELISA does not provide any knowledge about the location of the FAP activity, but by being much simpler to use and less dependent on complex machinery, the C3F assay could be a more optimal approach for FAP-related drug testing and for the more frequent monitoring of patients. Alternatively, there could be great potential in using the C3F assay to identify the patients most eligible to benefit from an FAPI-tracer PET/CT scan.

Both clinical cohorts were small and further investigations are needed. As such, the biomarker could be a useful tool in other diseases, as FAP has been shown to be upregulated and influential in multiple types of cancer as well as non-cancerous diseases including acute coronary syndrome, fibrosis, and arthritis [54,55,56,57]. For future studies, it would be interesting to investigate if C3F could show other biomarker properties like those of FAP protein quantification as multiple studies have shown an indication of its prognostic or predictive potential in multiple cancer types including renal, colorectal, hepatic, and lung cancer [25,58,59,60]. It is also of great interest to investigate the value of C3F as a predictive or pharmacodynamic biomarker for various FAP-targeting treatment procedures as this could be where the use of C3F will be applied in the future.

## 5. Conclusions

FAP activity can be quantified non-invasively (in serum) by the C3F ELISA. The C3F ELISA is targeting a peptide fragment of type III collagen, specifically released to circulation upon FAP cleavage of already partly degraded type III collagen, which indicates that the fragment is associated with high disease activity. When measured in cohorts of patients with NSCLC and SpA, two diseases that have been associated with FAP and high tissue turnover, we saw that C3F was significantly elevated compared to healthy controls, suggesting a potential use for the biomarker across disease with FAP activity.

## 6. Patents

A patent has been filed based on the invention presented in this study.

## Figures and Tables

**Table 1:** Patient demographics and clinical profile for cohort 1: patients with non-small cell lung cancer (NSCLC) and healthy individuals.

Clinical Variables	Patients with NSCLC (*n* = 109)	Healthy Individuals (*n* = 42)
Age (years)		
Median (min–max)	62 (37–81)	54 (37–69)
Sex, *n* (%)		
Male	77 (71%)	30 (71%)
Female	32 (29%)	12 (29%)
NSCLC subtype, *n* (%)		
Adenocarcinoma	66 (61%)	
Squamous cell carcinoma	43 (39%)	
Body mass index (BMI)		
Median (min–max)	26 (16–36)	
Stage		
3	40 (37%)	
4	69 (63%)	

**Table 2:** Patient demographics and clinical profile for cohort 2: patients with Spondyloarthritis (SpA) and healthy individuals.

Clinical Variables	Patients with SpA (*n* = 17)	Healthy Individuals (*n* = 19)
Age (years)		
Median (min–max)	49 (27–69)	51 (38–69)
Sex, *n* (%)		
Male	10 (59%)	11 (58%)
Female	7 (41%)	8 (42%)
SpA subtype, *n* (%)		
Axial spondyloarthritis	7 (41%)	
Psoriatic arthritis	10 (59%)	
Have received treatment, *n* (%)		
No	9 (53%)	
Yes	7 (41%)	
Unknown	1 (6%)	

**Table 3:** Summary of technical evaluation of C3F assay.

Test	Results
IC50	10.4 nM
Quantification range (LLOQ-ULOQ)	1.27–250 nM
Lower limit of detection	0.96 nM
Dilution recovery (1:2→1:4)	93%
Spiking recovery (serum in serum)	90%
Interference (hemoglobin, low/high conc.)	102%/106%
Interference (biotin, low/high conc.)	107%/109%
Interference (lipids, low/high conc.)	103%/108%
Inter-assay variation	13%
Intra-assay variation	7%
Freeze/thaw stability up to four cycles	84%

## Data Availability

All data that support the findings presented in this paper are available upon request from the corresponding author.
